# Total joint Perioperative Surgical Home: an observational financial review

**DOI:** 10.1186/2047-0525-3-6

**Published:** 2014-08-27

**Authors:** Darren R Raphael, Maxime Cannesson, Ran Schwarzkopf, Leslie M Garson, Shermeen B Vakharia, Ranjan Gupta, Zeev N Kain

**Affiliations:** 1 Department of Anesthesiology and Perioperative Care, University of California, 333 The City Boulevard West, Suite 2150, Orange, Irvine, California 92868, USA; 2 Department of Orthopedic Surgery, University of California, 101 The City Drive South Pavilion III, Building 29A Orange, Irvine, California 92868, USA

**Keywords:** Perioperative surgical home, Perioperative practice model, Perioperative care pathway, Total arthroplasty, Cost analysis, Cost variation

## Abstract

**Background:**

The numbers of people requiring total arthroplasty is expected to increase substantially over the next two decades. However, increasing costs and new payment models in the USA have created a sustainability gap. *Ad hoc* interventions have reported marginal cost reduction, but it has become clear that sustainability lies only in complete restructuring of care delivery. The Perioperative Surgical Home (PSH) model, a patient-centered and physician-led multidisciplinary system of coordinated care, was implemented at UC Irvine Health in 2012 for patients undergoing primary elective total knee arthroplasty (TKA) or total hip arthroplasty (THA). This observational study examines the costs associated with this initiative.

**Methods:**

The direct cost of materials and services (excluding professional fees and implants) for a random index sample following the Total Joint-PSH pathway was used to calculate *per diem* cost. Cost of orthopedic implants was calculated based on audit-verified direct cost data. Operating room and post-anesthesia care unit time-based costs were calculated for each case and analyzed for variation. Benchmark cost data were obtained from literature search. Data are presented as mean ± SD (coefficient of variation) where possible.

**Results:**

Total *per diem* cost was $10,042 ± 1,305 (13%) for TKA and $9,952 ± 1,294 (13%) for THA. Literature-reported benchmark *per diem* cost was $17,588 for TKA and $16,267 for THA. Implant cost was $7,482 ± 4,050 (54%) for TKA and $9869 ± 1,549 (16%) for THA. Total hospital cost was $17,894 ± 4,270 (24%) for TKA and $20,281 ± 2,057 (10%) for THA. In-room to incision time cost was $1,263 ± 100 (8%) for TKA and $1,341 ± 145 (11%) for THA. Surgery time cost was $1,558 ± 290 (19%) for TKA and $1,930 ± 374 (19%) for THA. Post-anesthesia care unit time cost was $507 ± 187 (36%) for TKA and $557 ± 302 (54%) for THA.

**Conclusions:**

Direct hospital costs were driven substantially below USA benchmark levels using the Total Joint-PSH pathway. The incremental benefit of each step in the coordinated care pathway is manifested as a lower average length of stay. We identified excessive variation in the cost of implants and post-anesthesia care.

## Background

Total knee arthroplasty (TKA) and total hip arthroplasty (THA) show high cost–utility, cost-effectiveness, and cost–benefit over other interventions [1-3]. However, the cost of care delivery in the USA has increased to the point that total arthroplasty (TA) is now the largest expenditure per procedure in Centers for Medicare and Medicaid Services (CMS)-provided interventions [4]. Coupled with declining reimbursement, hospitals have struggled to maintain profitability for these procedures. The passage of the Affordable Care Act and implementation of performance-based bundled payments threatens to exacerbate this sustainability gap if significant cost-control measures are not implemented. Furthermore, as the ‘baby boomer’ generation ages and obesity continues to rise in the general population, the demand for primary TKA and THA in the USA is expected to increase substantially over the next two decades [5,6].

It is well established that costs for TA during the initial hospital stay are mostly driven by three major factors: implant cost, hospital length of stay (LOS), and operating room (OR) cost [4,7-9]. Implant cost has increased sharply over the past two decades, with many new and more complex options brought to market. Implant cost minimization strategies may be found in the literature, including implant standardization, group purchasing, gain sharing, price ceilings, and the creation of a national joint database to track outcomes and inform purchasing decisions. LOS reduction efforts have examined modern surgical techniques and multimodal pain management, and described post-operative clinical pathways that employ early mobilization and rehabilitation as well as reduction of post-operative complications. OR cost reduction has focused mainly on surgical techniques and operational efficiency. These *ad hoc* interventions have been shown to reduce costs marginally in USA hospitals, but none has addressed the broader issue of a fragmented and inefficient perioperative system. The transition to performance-based bundled payments in the USA has illustrated the need for the adoption of perioperative practice models similar to those that have been in place in Europe for over a decade with proven financial benefits.

We submit that sustainable cost reduction lies only in a complete restructuring of how TA care is delivered. The Perioperative Surgical Home (PSH) is a recently proposed perioperative practice model in the USA. The goal of the PSH is to improve clinical outcomes while providing better perioperative service to patients at lower cost [10,11]. This model has been described as a ‘patient-centered and physician-led multidisciplinary and team-based system of coordinated care that guides the patient throughout the entire surgical experience.’ The first PSH program was implemented at UC Irvine Health in 2012 for all elective TKA and THA [12,13]. The intent of the program is to support the orthopedic surgeon, ensure adherence to mutually agreed-upon protocols, and manage medical issues that arise during the episode of care. The surgeon’s role as ultimate decision-maker is maintained. A major aim of the Total Joint-PSH protocol is to reduce variation in care delivery, which will in turn reduce cost and improve outcomes [4,12-15]. This observational study examines the cost and cost variation associated with the Total Joint-PSH, and compares to reported benchmarks.

## Methods

We performed an observational cost analysis for patients undergoing primary unilateral elective THA or TKA under the Total Joint-PSH model at UC Irvine Health between October 1, 2012 and September 30, 2013. Institutional review board approval was obtained with the purpose of analyzing and reporting our results, and patient consent was waived (IRB HS#2012-9273). The implementation of the Total Joint-PSH program at our institution has been described in detail elsewhere [12,13].

### Implementing the Total Joint-PSH program

The Total Joint-PSH program was created prior to reestablishment of an arthroplasty center at UC Irvine Health in 2012. The lack of an existing program allowed all stakeholders (including orthopedic surgeons, anesthesiologists, acute pain physicians, nurses, rehabilitation specialists, and hospital administrators) to have a voice in its design and implementation. All team members were trained in Lean Six Sigma (LSS), and agreed to adhere to the concepts of standardization and reduced variability. The goal of this process was to integrate four distinct perioperative phases: pre-operative, intra-operative, post-operative and post-discharge. A value stream map (flow diagram documenting in high detail every step of the process) was created for each perioperative phase. The pre-operative process incorporates expectation management, early discharge planning, protocol-driven health risk assessment, and medical optimization. Standardized anesthetic, nursing, and surgical care protocols, as well as Goal Directed Fluid Therapy (GDFT) underpin the intra-operative component. Post-operative management provides for multimodal analgesia, a targeted recovery plan, early ambulation, nutrition management, and prompt rescue from complications. Post-discharge care begins in the hospital with coordinated transition to an appropriate rehabilitation setting. Once the perioperative pathways were fully vetted, the Total Joint-PSH program was officially launched on October 1, 2012.

### Cost analysis

#### ***Per diem cost analysis***

The direct cost of materials and services (excluding professional fees and implants) provided during each hospital day of care were obtained from the UC Irvine Health financial decision support office. Data were provided for a randomly chosen index sample (n = 29 or 14%) of patients who had undergone unilateral primary arthroplasty following the Total Joint-PSH pathway. Data from this sample were used to determine average *per diem* cost for each day of admission. Components of *per diem* cost are presented in Table 1. *Per diem* cost for post-operative day (POD) 0 included costs incurred during pre-operative health risk assessment and optimization in the operating room (OR) and in the post-anesthesia care unit (PACU). The cost of pre-operative orthopedic clinic professional visits was not included. Costs incurred during POD 1 to 3 included room and board as well as all activities related to recovery and discharge planning. The previously reported average LOS for the Total Joint-PSH was 2.7 ± 0.64 days for TKA and 2.6 ± 0.67 for THA [12], thus a conservative value of 3 days LOS was used to calculate total *per diem* cost.

**Table 1:** **Components of *per diem *** **cost**

**Time-based**	**Non time-based**
Operating room time	Equipment
Post-anesthesia care unit time	Materials
	Laboratory
	Pharmacy
	Physical therapy
	Room and board

#### ***Specialized orthopedic materials and implant cost analysis***

All specialized orthopedic materials and implants used in the OR were recorded by nursing staff intra-operatively, and verified by the revenue audit department. Data from all patients (n = 206) undergoing elective primary unilateral TKA and THA were used to examine the cost of orthopedic materials and implants. The average cost of orthopedic materials and implants was calculated using the acquisition cost.

#### ***Total cost analysis***

Total cost for primary TKA and THA performed as part of the Total Joint-PSH program was calculated as the sum of the total *per diem* cost, orthopedic materials cost, and implant cost. Total cost for was calculated for all cases.

#### ***Determination of OR and PACU cost per minute***

The cost of OR time was calculated in the following manner. Aggregate direct cost data for the index patient sample was obtained from the decision support office. OR-related costs (excluding implants) were identified. Materials typically used during the first 30 minutes of OR time were also identified. Two average total costs were calculated, one for the first 30 minutes and one for costs incurred thereafter. Total OR time was obtained by database query of the intra-operative electronic medical record (EMR) (Surgical Information Systems, LLC, Alpharetta, GA, USA). Using these data, a cost per minute was calculated for the first 30 minutes and for subsequent OR time. An analogous process was performed to obtain cost per minute values for PACU time.

#### ***OR and PACU time variation and cost analysis***

Total OR and PACU times were determined by database query of the intra-operative and post-operative EMR for all patients undergoing elective unilateral primary TKA and THA. In-room to incision time and surgical time were also obtained. Using the calculated cost per minute values, a time-based cost was determined for the components of each case.

### Data analysis

Variation in cost was assessed using coefficient of variation (defined as SD/mean). Data are presented as mean ± SD (coefficient of variation). All statistics were performed using SPSS software version 11.0 (SPSS Inc., Chicago, IL, USA).

## Results

In total, 206 (n = 129 for TKA and n = 77 for THA) sequential patients undergoing unilateral primary TA were enrolled in the Total Joint-PSH protocol. Demographics are presented in Table 2.

**Table 2:** **Demographics of the included patients**^**a**^

	**TKA (n = 129)**	**THA (n = 77)**
Age	65 ± 10.53	64 ± 13.82
BMI	30.7 ± 5.7	28.5 ± 7.2
Spinal anesthesia	61%	57%
General anesthesia	39%	43%
ASA grading		
I	0%	1%
II	15%	21%
III	79%	73%
IV	6%	5%

ASA, American Society of Anesthesiologists Physical Status; BMI, body mass index; THA, total hip arthroplasty; TKA, total knee arthroplasty.

^a^Data are expressed as mean ± SD.

### Total cost analysis

**Figure 1:**
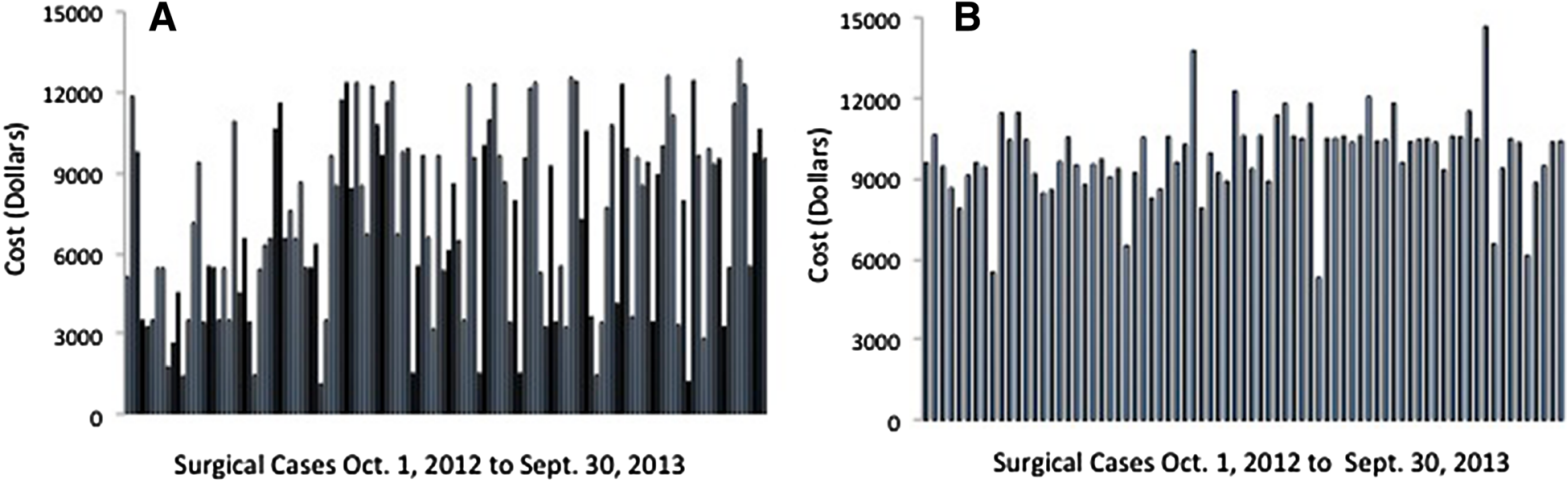
**Implants cost. (A)** total knee arthroplasty; (**B)** total hip arthroplasty.

A summary of costs (*per diem* cost, orthopedic materials and implants cost, and total calculated cost) is presented in Table 3. Individual cost of implants for all cases is presented in Figure 1.

**Table 3:** **Summary of costs**^**a**^

**Costs**	**TKA**	**THA**
*Per diem* cost (LOS = 3 days)	$10,042 ± 1305 (13%)	$9,952 ± 1294 (13%)
Orthopedic materials and implants	$7,482 ± 4050 (54%)	$9,869 ± 1549 (16%)
Total cost	$17,524 ± 4255 (24%)	$19,821 ± 2018 (10%)

LOS, length of stay; THA, total hip arthroplasty; TKA, total knee arthroplasty.

^a^Data are expressed as mean ± SD (coefficient of variation).

### Time-based cost analysis

**Figure 2:**
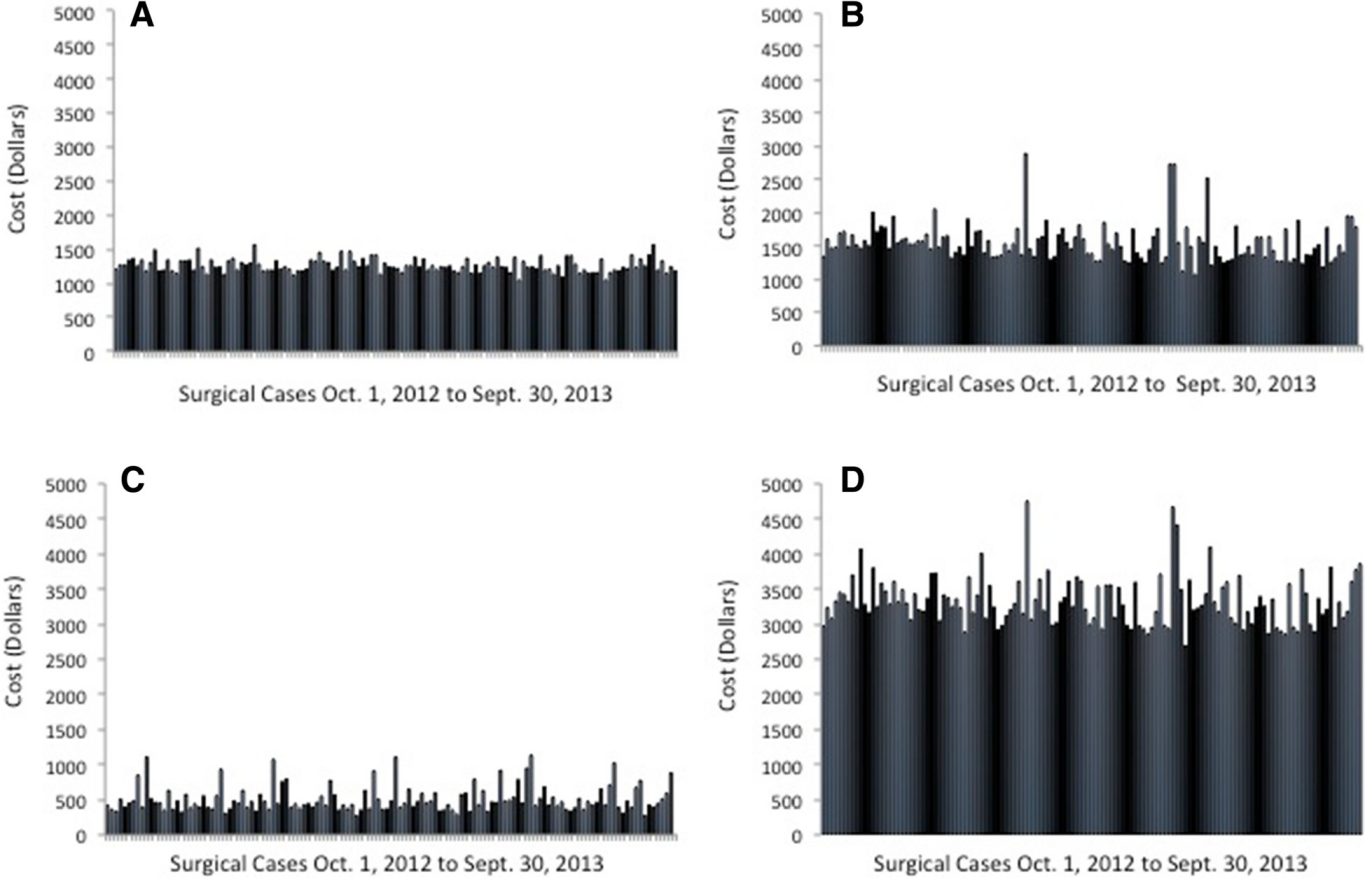
**Surgical time cost for total knee arthroplasty. (A)** In-room to incision time cost; **(B)** surgery time cost; **(C)** post-anesthesia care unit (PACU) time cost; and **(D)** total operating room (OR) and PACU time cost.

**Figure 3:**
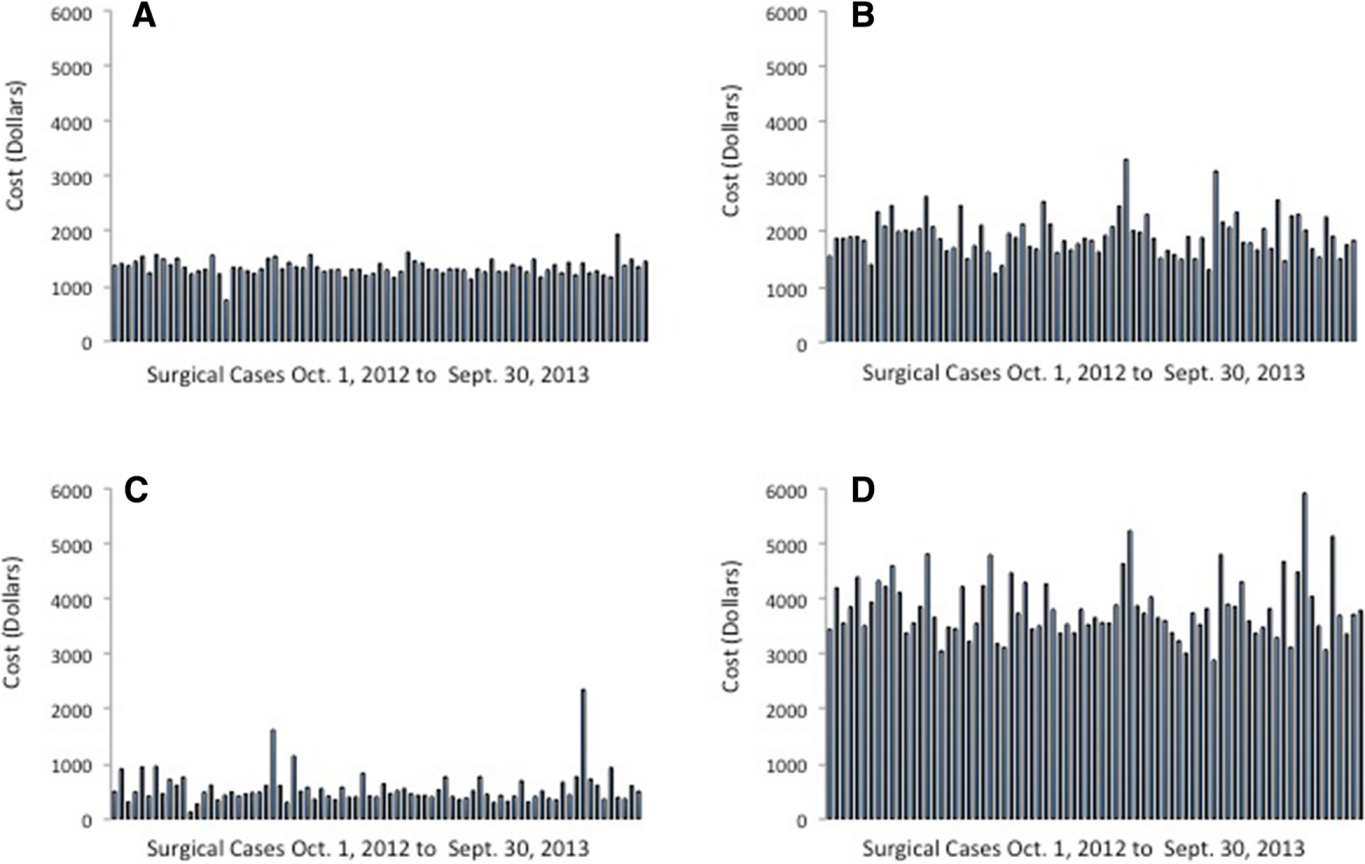
**Surgical time cost for total hip arthroplasty. (A)** In-room to incision time cost; **(B)** surgery time cost; **(C)** post-anesthesia care unit (PACU) time cost; **(D)** total operating room (OR) and PACU time cost.

A summary of OR time costs is presented in Table 4. Individual case costs (in-room to incision time cost, surgery time cost, PACU time cost, and total OR and PACU time cost) for TKA and THA are presented in Figure 2 and Figure 3, respectively.

**Table 4:** **Summary of OR time costs**^**a**^

	**TKA**	**THA**
In-room to incision time cost	$1,263 ± 100 (8%)	$1,341 ± 145 (11%)
Surgery time cost	$1,558 ± 290 (19%)	$1,930 ± 374 (19%)
PACU time cost	$507 ± 187 (36%)	$557 ± 302 (54%)
Total OR & PACU time cost	$3,329 ± 350 (11%)	$3,828 ± 559 (16%)

OR, operating room; PACU, post-anesthesia care unit; THA, total hip arthroplasty; TKA, total knee arthroplasty.

^a^Data are expressed as mean ± SD (coefficient of variation).

## Discussion

Our analysis shows a low total cost for unilateral primary TKA and THA in the setting of the first Total Joint-PSH. Prior to implementation of the Total Joint-PSH program, our institution had no active arthroplasty program. This study is observational in nature, and no comparison with prior costs was made. We sought instead to benchmark our cost data against figures reported in the literature. A recent retrospective study of primary total joint arthroplasty (TJA) found an average hospital cost (episode cost excluding implants) of $17,588 for TKA and $16,267 for THA. Hospital episode cost for the Total Joint-PSH patient at our institution ($10,042 ± 1305 for TKA and $9,952 ± 1294 for THA) was found to be significantly below this benchmark (Table 5). The reduced LOS in our institution (4 versus 3 days) is a major factor in this comparative cost reduction. We contend that the incremental benefit of each step in the Total Joint-PSH coordinated care pathway is manifested as a low average LOS (rapid recovery). Cost savings attributed to reduced LOS must be considered in the context of equivalent outcomes, as complications can contribute substantially to the overall cost of arthroplasty. We have previously reported low complication and re-admission rates for patients in the Total Joint-PSH compared with published data [12], but the contribution of complications to overall cost of care was not considered in this analysis.

**Table 5:** **Benchmark cost comparison: average hospital cost excluding implants**^**a**^

	**Total Joint-PSH**	**Benchmark [**[16]**]**
TKA	$10,042 ± 1,305	$17,588
THA	$9952 ± 1,294	$16,267

PSH, perioperative surgical home; THA, total hip arthroplasty; TKA, total knee arthroplasty.

^a^Data are expressed as mean ± SD.

The second largest cost driver was identified as cost of implants $7,482 ± 4,050 (TKA) and $9,869 ± 1,549 (THA). Implant cost was recently examined in the literature, and found to range from $1,797 to $12,093 (TKA) and $2,392 to $12,361 (THA) [9]. Implant cost at our institution was within this benchmark range. However, the cost of implants was the largest source of cost variation in the Total Joint-PSH, at 54% (TKA) and 16% (THA). Variation in implant cost is appropriate as a reflection of patient demographics and underlying conditions, particularly in the setting of a tertiary care academic center. However, a component of this variation can be attributed to other factors. UC Irvine Health is currently engaged in an initiative to reduce implant cost variability and overall implant cost.

Operating room and PACU cost was determined using a time-based cost analysis. It has been argued that reduction in OR time does not result in cost savings unless enough time is saved to add an additional case during regular operating hours. This reasoning presumes all OR costs are fixed; however, a number of OR resources (such as staffing) are variable direct costs that can be reallocated if OR time is reduced. Determination of the time-based cost can help to identify areas for OR cost savings and illustrate the importance of operational efficiency. We examined variation in terms of cost rather than time because our model assigns a higher cost to the initial 30 minutes of OR and PACU times. In our analysis, variation in OR time cost for the Total Joint-PSH was found to be low. Cost associated with in-room to incision time varied by 8% (TKA) and 11% (THA). We attribute this low variation to a well-defined, time-limited decision tree for conversion of difficult neuraxial to general anesthesia. Cost associated with surgery time varied by 19% (TKA and THA). Relatively low variation in these parameters enables predictable optimization of OR scheduling. Excessive variation of 36% (TKA) and 54% (THA) was found in the PACU time cost analysis. The data showed several outliers, which upon initial investigation were attributable to limited availability of beds in the orthopedic ward at the desired time of PACU discharge. This finding has alerted the Total Joint-PSH team to the need for further study and an LSS analysis of this process step.

Minimizing direct hospital costs without improvement in care or outcomes may simply shift cost to the post-discharge arena. The cost of orthopedic rehabilitation hospital care, home-based care, emergency room visits, complications, and readmissions must be closely tracked. A recent study of TJA costs found that post-discharge payments accounted for an average of 36% of total episode of care payments [16]. The current observational cost analysis of the Total Joint-PSH cohort did not consider post-discharge care, and some component of cost shifting may exist. We recognize this as a challenging and pressing area of future study.

The PSH is a care delivery model that has been endorsed by the American Society of Anesthesiologists (ASA), with the goal of improving clinical outcomes while providing better perioperative service at lower cost. Future studies will be geared toward comparing the PSH with other models of care.

## Conclusions

We found that direct hospital costs can be driven substantially below benchmark levels using the Total Joint-PSH pathway, and suggest that implementation of a PSH model of care could help institutions to better control process costs and identify unwarranted costs. In the case of the Total Joint-PSH, we have identified an opportunity to decrease variation in the cost of implants and the cost of PACU time.

## Abbreviations

ASA: American Society of Anesthesiologists; BMI: Body mass index; CMS: Centers for Medicaid and Medicare; EMR: Electronic medical record; GDFT: Goal directed fluid therapy; LOS: Length of Stay; LSS: Lean Six Sigma; OR: Operating room; PACU: Post-anesthesia care unit; POD: Post-operative day; PSH: Perioperative Surgical Home; TA: Total arthroplasty; THA: Total hip arthroplasty; TJA: total joint arthroplasty; TKA: Total knee arthroplasty.

## Competing interests

RS performs consulting work for Smith & Nephew, owns stock in Gauss Surgical and Pristine and does research for Pricria; LMG owns stock warrants in Pristine; and SBV has received a fellowship grant from The Center for Healthcare Quality and Innovation for Urology Surgical Home. The other authors have no competing interests to declare.

## Authors’ contributions

DR participated in study design, was responsible for data acquisition, performed statistical analysis and figure design, and authored the manuscript; MC participated in study design, performed statistical analysis and figure design, and co-authored the manuscript; RS participated in study design, contributed to data analysis, and co-authored the manuscript; LG participated in study design, contributed to data acquisition, and co-authored the manuscript;. SV participated in study design and co-authored the manuscript; MC participated in study design and co-authored the manuscript; and ZK participated in study design, contributed to data analysis and co-authored the manuscript. All authors read and approved the final manuscript.
